# 

**Published:** 1873-07

**Authors:** 


### CONTENTS.

**COMMUNICATIONS.**

PAGE.

Some Obsolete Aphorisms. Ben H. Pratt, A. M...... 139
About Homeopathy. P. H. Hayes, M. D................ 141
Nursing. B. B...................................... 143

**CORRESPONDENCE.**

Ten Letters, with answers.......................... 151

**CLIPPINGS.**

The Betel Nut...................................... 144
Transplanting Hair................................. 144

**EDITORIAL.**

PAGE

Medical Items and News............................. 145
Household Hints and Recipes........................ 154
Keep Cool.......................................... 158
Health Journals.................................... 158
Spectacles......................................... 159
Chit-Chat.......................................... 162
Books and Periodicals.............................. 164

